# Magnetic Resonance Spectroscopy, Positron Emission Tomography and Radiogenomics—Relevance to Glioma

**DOI:** 10.3389/fneur.2018.00033

**Published:** 2018-02-05

**Authors:** Gloria C. Chiang, Ilhami Kovanlikaya, Changho Choi, Rohan Ramakrishna, Rajiv Magge, Dikoma C. Shungu

**Affiliations:** ^1^Department of Neuroradiology, Weill Cornell Medical College, New York, NY, United States; ^2^Radiology, Advanced Imaging Research Center, University of Texas Southwestern Medical Center, Dallas, TX, United States; ^3^Department of Neurological Surgery, Weill Cornell Medical College, New York, NY, United States; ^4^Department of Neurology, Weill Cornell Medical College, New York, NY, United States

**Keywords:** radiogenomics, glioma, magnetic resonance spectroscopy, positron-emission tomography, neurooncology, neuroradiology

## Abstract

Advances in metabolic imaging techniques have allowed for more precise characterization of gliomas, particularly as it relates to tumor recurrence or pseudoprogression. Furthermore, the emerging field of radiogenomics where radiographic features are systemically correlated with molecular markers has the potential to achieve the holy grail of neuro-oncologic neuro-radiology, namely molecular diagnosis without requiring tissue specimens. In this section, we will review the utility of metabolic imaging and discuss the current state of the art related to the radiogenomics of glioblastoma.

## Introduction

The role of neuro-radiology in the management of glioma has shown a stepwise evolution over time. As the field of neuro-oncology has progressed, neuro-radiology has also made significant strides. A significant advance in the last decade has been the emergence of imaging modalities that help distinguish tumor from treatment effect. Moreover, the emerging field of radiogenomics wherein imaging features are correlated with genomic attributes of disease promises to be a significant boon for patients. Such data could serve as reliable biomarkers of disease where treating physicians could use the information to guide treatments in the absence of new pathologic specimens. In this review, we discuss metabolic imaging and radiogenomics and assess the future of these modalities, respectively.

## Magnetic Resonance Spectroscopy

**Figure 1:**
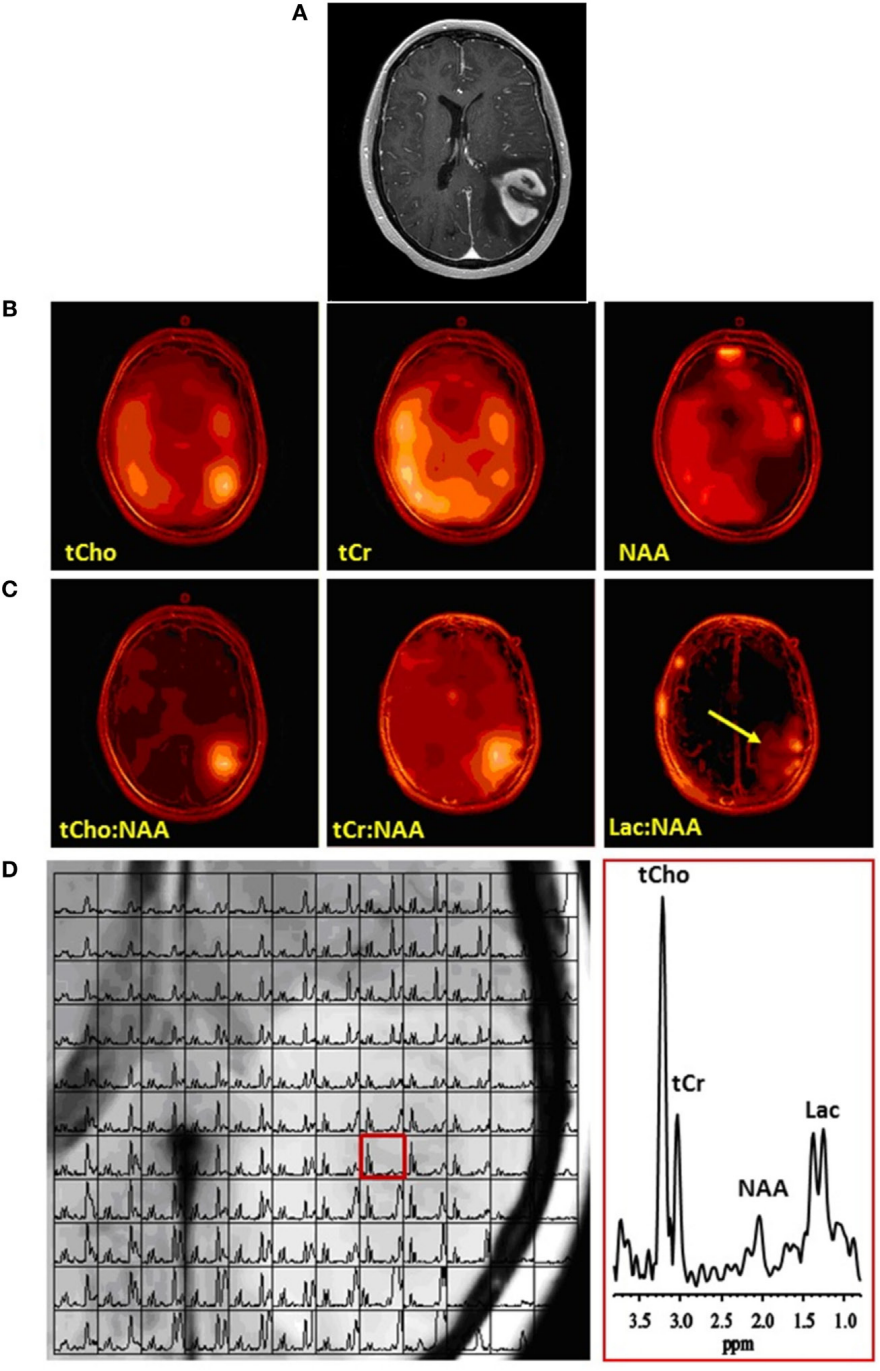
Multivoxel MRS of a glioblastoma. **(A)** Contrast-enhanced axial MR image demonstrating an enhancing mass in the left parietal lobe. **(B)** Corresponding multivoxel or spectroscopic imaging data displayed as color maps that show the spatial distributions of (left-to-right) total choline (tCho), total creatine (tCr), and *N*-acetylaspartate (NAA). Note marked elevation of tCho and decrease of NAA at the location of the tumor. **(C)** The same spectroscopic imaging data displayed as color maps of metabolite ratios: (left-to-right) tCho:NAA, tCr:NAA, and lactate or Lac:NAA. Because NAA decreased while tCho and Lac increased and tCr did not change appreciably, the maps of metabolite ratios to NAA result in enhancement and better definition of metabolite signals at the location of the tumor. Arrow on Lac:NAA map shows Lac to extend beyond the tumor margins. **(D)** (Left) Grid plot of the multivoxel data overlaid on the corresponding MR image of the tumor, and (right) a spectrum in a single voxel extracted from the multivoxel data at the location of the tumor (red box on left panel), showing clearly elevated tCho and Lac and decreased NAA resonances. No copyright permissions were required for use of these images.

Proton magnetic resonance spectroscopy is a non-invasive technique that enables measurement of levels of brain metabolites. *N*-acetylaspartate (NAA) is widely used as a marker of neuronal integrity that is decreased in tumors. Total choline (tCho) is considered a marker of neoplastic proliferation and is important in cell membrane biosynthesis and turnover. Total creatine (tCr) is an energy metabolite that may vary based on tumor type and grade. Lactate is the end-product of glycolytic metabolism and is often increased in high-grade gliomas. Finally, measurable lipid, specifically triglycerides, is considered a marker of necrosis. Single-voxel MRS methods have been used to compare levels of these metabolites in the tumor and in adjacent or contralateral normal brain. Also available are 2-dimensional multi-slice (1) and 3-dimensional (2) multi-voxel spectroscopic imaging techniques (Figure 1), which have been used to address tumor heterogeneity, by examining different levels of these metabolites in different portions of the tumor and the surrounding brain.

Both single-voxel and multi-voxel MRS have been widely used for grading gliomas preoperatively. Commonly, ratios of metabolite peak areas are reported, with tumoral regions demonstrating higher tCho:NAA and tCho:tCr ratios, as well as lower NAA:tCr ratios, compared to non-tumoral regions. A meta-analysis of 30 studies, which included studies using both single- and multi-voxel MRS at 1.5 and 3.0 T, reported a pooled sensitivity and specificity of 80% (range 67–100%) and 76% (range 63–100%) for Cho:NAA, 75% (range 49–100%) and 60% (range 3–100%) for tCho:tCr, and 71% (range 30–100%) and 70% (range 40–100%) for NAA:tCr (3). Overall accuracy in differentiating high- and low-grade gliomas was greatest with the tCho:NAA, with an area under the curve of 0.87. Another study that evaluated molar or “absolute” metabolite concentrations rather than peak area ratios found that a maximum tCho concentration cutoff of 2.02 mmol/L resulted in a sensitivity of 86% and specificity of 78%, and a mean tCho concentration cutoff of 1.52 mmol/L resulted in a sensitivity of 78% and specificity of 63% (4).

There are several limitations of MRS. First, MRS requires relatively long scan times, particularly for multivoxel MRS, due to the millimolar-range concentration of the metabolites and the limited detection sensitivity of the technique. Second, MRS requires additional post-processing time and expertise for multi-voxel and several specialized single-voxel techniques. Finally, voxel placement is user-dependent and requires avoiding contamination of metabolites of interest with lipid signal from the calvarial marrow, particularly at the skull base, and soft tissues of the scalp.

The ^1^H MRS technique is also useful in differentiating between recurrent tumor and radiation necrosis in posttreatment glioma surveillance. As in the pretreatment setting, recurrent tumors demonstrate higher tCho:NAA and tCho:tCr, as well as lower NAA:tCr, compared to radiation necrosis (5–8). Sensitivity ranges from 36 to 94%, specificity 55–100% (5, 8–14), with accuracy as high as 97% (7, 8). A systematic review of 17 prior studies found a weighted average sensitivity of 86% and specificity of 80% (9). Another study reported the added utility of the tCho:tCr peak area using multivoxel MRS, resulting in an area-under-the-curve of 0.91, sensitivity 96%, specificity 83%, and the tCho:NAA peak-height (area-under-the-curve 0.91, sensitivity 91%, specificity 83%), which performed better than single-voxel MRS (10).

**Figure 2:**
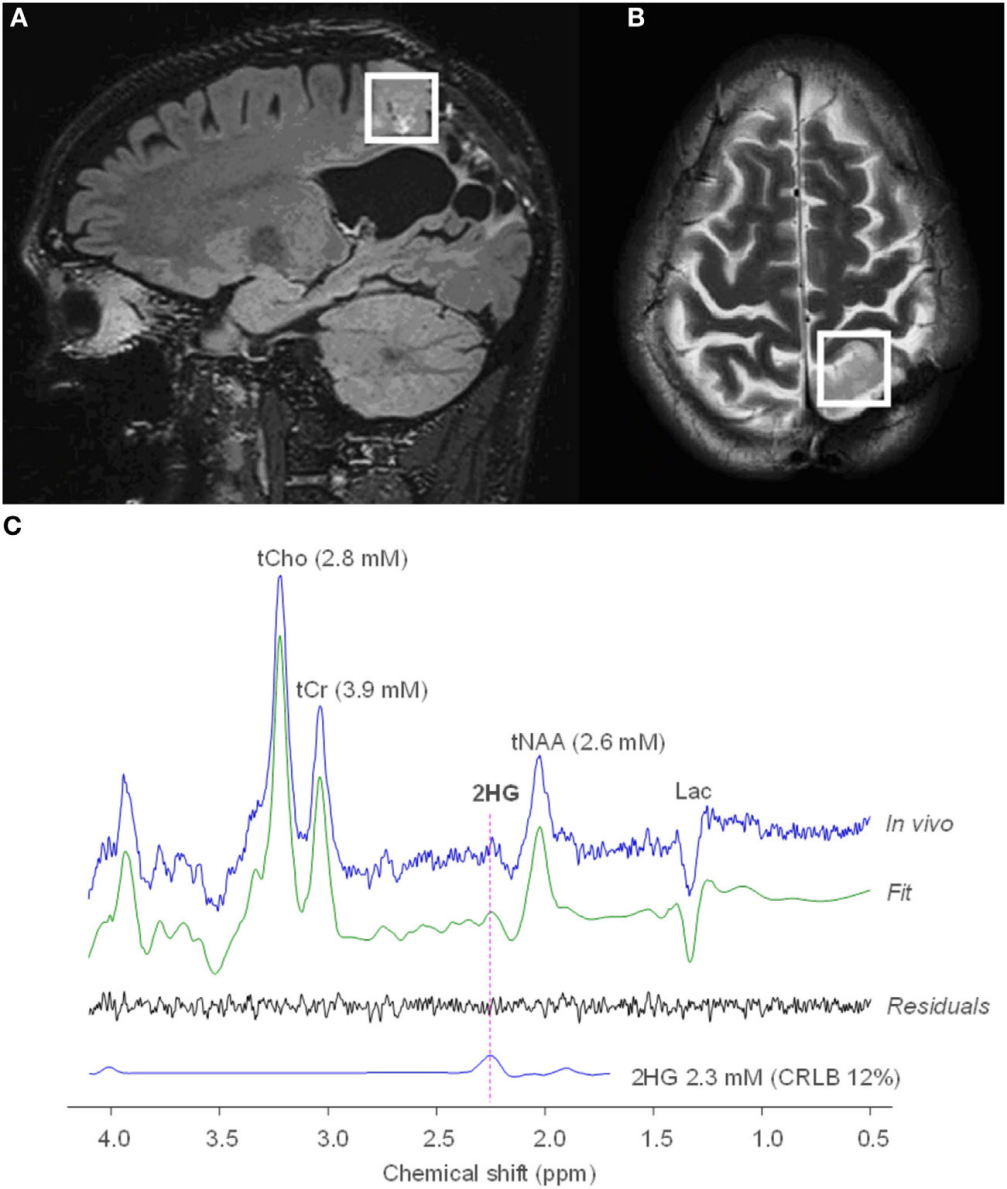
MRS of R-2-hydroxyglutarate (2HG) in an oligodendroglioma. Sagittal 3D T2 FLAIR **(A)** and axial T2-weighted **(B)** imaging shows a T2-hyperintense mass in the left parietal lobe. Single voxel MRS in the tumoral region demonstrates elevated 2HG concentration **(C)**, compatible with an isocitrate dehydrogenase-mutated glioma. No copyright permissions were required for use of these images.

Recent literature has focused on the importance of isocitrate dehydrogenase (IDH) mutations in glioma prognostication (11, 12). Gliomas that harbor an IDH mutation accumulate the oncometabolite, R-2-hydroxyglutarate (2HG), which can be detected by ^1^H MRS (Figure 2) (13). A recent longitudinal study in 136 glioma patients showed that 2HG MRS provides a noninvasive means of monitoring IDH-mutated tumor cells during disease progression and treatment (14). In a prospective longitudinal cohort of 89 patients (15), 2HG MRS was found to have a high test–retest correlation of 0.98 and had a sensitivity of 100% and specificity of 88% using a threshold of 1 mmol/L. A study using a similar cutoff of 1.489 mmol/L for 2HG MRS found a sensitivity of 100% and specificity of 72%, with higher concentrations predicting better survival (16). The use of 2HG MRS in the preoperative setting may enable a diagnosis of IDH mutant glioma more rapidly and practically than genetic testing and may thus serve as a biomarker for selecting patients who may be ideal candidates for neoadjuvant or adjuvant IDH-inhibitor trials. This technique could also serve as a noninvasive marker for monitoring tumor volume changes throughout the course of the trials, complementing conventional MR imaging with highly specific information on the status of tumor cells.

Finally, alterations in tumor metabolism are being exploited for therapeutic approaches. For example, a ketogenic diet has been reported as a potential metabolic therapy for gliomas, since tumor cells may lack the flexibility to use ketone bodies during glucose restriction whereas normal brain cells can. MRS may thus detect elevated levels of ketone bodies in the brain, either due to increased uptake of ketone bodies in the brain or decreased utilization by tumor cells (17). Furthermore, increased expression of lactate dehydrogenase A has been described in glioblastoma, resulting in increased production of lactate, termed the Warburg effect (18). Using ^13^C MRS and hyperpolarization of ^13^C-labeled pyruvate, we can now monitor conversion to ^13^C-labeled lactate in real time. An increase in the hyperpolarized lactate-to-pyruvate ratio has been shown in murine models of glioblastoma, and a decrease in this ratio has been shown to follow treatment with inhibitors of the phosphoinositide 3-kinase/mammalian target of rapamycin (PI3K/mTOR) and temozolomide (19–22). As a first-in-man study in a small cohort of prostate cancer patients demonstrated the safety and feasibility of using hyperpolarized ^13^C MRS in cancer surveillance (23), more clinical trials in gliomas will likely be underway.

The role of MRS in glioma management has transitioned from identifying a classic tumor signature, i.e., an elevated choline-to-NAA ratio, to identifying specific oncometabolites, such as 2HG in IDH-mutated gliomas. As future glioma therapies target specific cancer metabolic pathways, incorporating the imaging specificity afforded by hyperpolarized ^13^C MRS into routine multimodal imaging surveillance will be of utmost importance.

## Positron Emission Tomography (PET)

2-(^18^F) fluoro-2-deoxy-d-glucose (FDG) PET has been widely used in the imaging of brain tumors. As a glucose analog, FDG is actively transported into the cell and phosphorylated by hexokinase in the glycolytic pathway. As a result, the FDG is trapped within the cell and can be imaged with uptake on PET being a proxy for glucose utilization and metabolic activity. High-grade brain tumors, being metabolically active, can demonstrate FDG uptake that is greater than normal surrounding brain regions, providing a means for non-invasive tumor diagnosis and monitoring. The commonly used metric to assess metabolic activity is the standardized uptake value (SUV), a ratio of the concentration of radioactivity in tissue to the injected dose per kilogram of the patient’s body weight. The SUV within a region-of-interest placed over the tumor can then be compared to a reference region, providing a semiquantitative measure of metabolic activity; either normal-appearing white matter or gray matter is typically used as a reference region.

This difference in metabolic activity has been widely utilized in the pretreatment setting to differentiate low and high-grade gliomas. Quantitatively, an SUV ratio of tumor to gray matter of greater than 0.6–0.7 and an SUV ratio of tumor to white matter of greater than 1.2–1.5 were suggestive of high-grade tumors (24–26). Posttreatment, the primary role of FDG-PET is to differentiate metabolically active tumor from treatment-related changes, such as radiation necrosis. As with pretreatment glioma grading, a visual rating scale, such as the Schifter metabolic grading scale (27), is commonly used to compare FDG uptake within a new or enlarging enhancing mass and normal white or gray matter. Although this grading scale can be easily and rapidly used in clinical practice, without the requirement of advanced and often time-consuming postprocessing, its reproducibility and accuracy have been questioned, with reported sensitivities ranging from 75 to 86% and specificities ranging from 22 to 95% (28–33). Furthermore, a meta-analysis of 26 studies reported a pooled sensitivity of 77% and specificity of 78% (34). Quantitative approaches for differentiating tumor from treatment-related changes have also been reported, with sensitivities ranging from 50 to 100% and specificities ranging from 65 to 100% (24, 31, 35–41). Reported optimal SUV cutoff ratios have ranged from 0.5 to 1.05 using normal gray matter as a reference region, 1.3 to 1.5 using normal white matter as a reference, and 1.2 to 1.35 using a mirror-image location as a reference. Finding more specific PET tracers to differentiate tumor from treatment-related changes is an area of active research.

In addition to grading gliomas and differentiating tumor from radiation necrosis, metabolic activity on FDG-PET has also been found to be prognostic. In a large cohort of 331 patients with low- and high-grade gliomas, Padma et al. (42) found that a metabolic grading scale accurately predicted overall survival. In a study of patients about to undergo treatment with bevacizumab and irinotecan for recurrent high-grade glioma, the maximum tumor SUV and the ratio of tumor SUV to contralateral normal cortex were both predictive of progression-free survival and overall survival on pretreatment FDG-PET (35). Posttreatment, both FDG-PET uptake (43, 44) and metabolic tumor volumes (45) have been reported to be prognostic. Further work may identify meaningful ways of combining FDG-PET and MR imaging variables with genetic/molecular markers for improved prognostication among patients with brain tumors.

Due to the lack of specificity of FDG-PET, particularly in the posttreatment setting, newer PET agents have been developed and reported. For example, amino acid analogs have been studied, since they are taken up at greater rates by amino acid transporters in tumors compared to non-tumor tissue. One of the earliest amino acid tracers reported in brain tumor imaging was l-methyl-^11^C-methionine (^11^C-MET). Pirotte et al. (46) found that ^11^C-MET PET was more accurate than FDG-PET in guiding tumor biopsies. ^11^C-MET PET was also reported to differentiate between low- and high-grade gliomas (24, 47, 48), differentiate grade 2 and grade 3 gliomas (49), correlate with proliferation (48) and vascularity (47), predict progression-free survival (49), and differentiate between recurrent tumor and radiation necrosis (50–52). However, a meta-analysis found that ^11^C-MET PET in the posttreatment setting remained less accurate than MR perfusion (53), with false positives seen with inflammation, necrosis, and hematomas (47, 48, 51). Due to the short half-life of ^11^C-MET, fluorinated amino acid analogs, i.e., O-2-^18^F-fluoroethyl-l-tyrosine and 3,4-dihydroxy-6-^18^F-fluoro-l-phenylalanine were developed and used in tumor imaging. Both the pretreatment sensitivity and specificity for detecting tumor have been reported to be 88%, with accuracy increasing to 97% with the addition of MR spectroscopy (54). Similarly, ^18^F-FET time–activity curves were reported to be only 67% accurate in differentiating low- and high-grade tumors, reaching 100% specificity after combination with ADC histogram analysis (55).

Posttreatment (18), F-FET has been found to be more sensitive and specific than conventional MRI in differentiating recurrent tumor from radiation necrosis, with sensitivities of 100% and specificities of 93–100% (56, 57). Tumor-to-brain ratios and textural features of ^18^F-FET have also been reported to differentiate pseudoprogression from true tumor progression, even more than 3 months after completion of radiochemotherapy (18, 58, 59). F-FDOPA PET can similarly detect tumor with higher sensitivity and specificity than FDG-PET, as well as differentiate tumor from radiation necrosis (60, 61). However, it has limited utility in differentiating low- and high-grade tumors (60). More recently, trans-1-amino-2-[18F]-fluorocyclobutane-carboxylic acid (^18^F-FACBC) has been reported to have a higher maximum SUV and tumor-to-background ratio than ^11^C-MET and ^18^F-FET (62). Finally, PET imaging with the radiotracer, alpha-[(11)C]-methyl-l-tryptophan, was reported to predict the location of subsequent tumor progression (63) and sites of radiation failure when used in treatment planning (64, 65). Prospective trials are ongoing to determine if adding amino-acid PET to MRI guidance for treatment planning improves progression-free survival in recurrent glioblastoma (66). These amino acid PET tracers remain investigational and more studies need to be done to determine whether they should be integrated into routine clinical practice.

Finally, several additional investigational PET tracers warrant discussion. The first, 3′-deoxy-3′[^18^F]-fluorothymidine accumulates within the cell after phosphorylation by thymidine kinase and is a marker of cell proliferation and DNA synthesis. As might be expected, ^18^F-FLT PET has been found to better differentiate between low- and high-grade gliomas (67, 68) than MR perfusion or MRS (68), and it demonstrates a stronger correlation with markers of proliferation, tumor progression, and overall survival than FDG-PET (67). However, there is less overall uptake than FDG-PET (67). Second, PET imaging using the tracer, ^18^F-fluoromisonidazole, has been reported as a quantitative measure of tumor hypoxia in glioblastomas, which may accelerate angiogenesis and shorten survival (69). Finally, specific PET tracers provide a non-invasive means of monitoring specific proteins important in tumor angiogenesis and invasion. Examples include ^68^gallium-labeled tracers targeting prostate-specific membrane antigen (18, 70), F-2-fluoropropionyl-labeled PEGylated dimeric arginine-glycine–aspartic acid (RGD) peptide targeting integrin α_vβ3_ (71), and ^89^Zr-labeled p-isothiocyanatobenzyl-desferrioxamine, targeting membrane-type 1 matrix metalloproteinase, important for tumor invasion (72).

**Figure 3:**
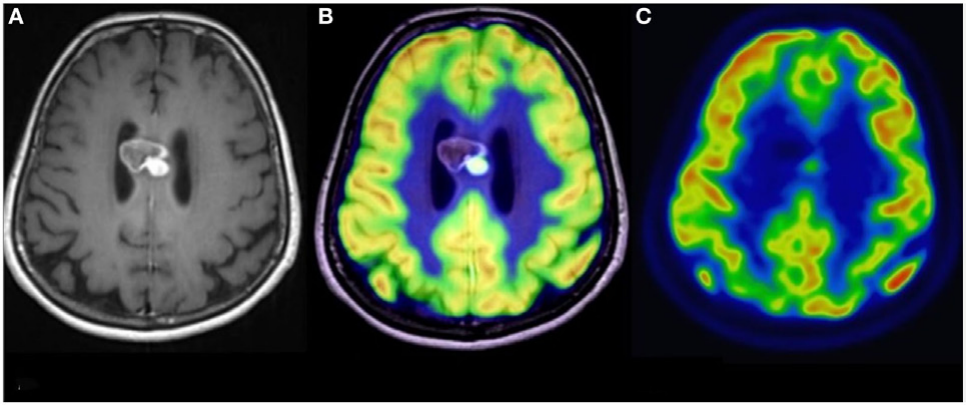
Simultaneous fluoro-2-deoxy-d-glucose (FDG) positron emission tomography (PET)-MRI of a glioblastoma. **(A)** Contrast-enhanced axial MR image demonstrating a heterogeneously enhancing mass in the corpus callosum. **(B)** Co-registered PET-MR image accurately demonstrating that the focus of elevated FDG avidity corresponds to the solidly enhancing component. **(C)** FDG PET image, without co-registration with MRI, demonstrates a focus of elevated FDG-avidity. No copyright permissions were required for use of these images.

**Figure 4:**
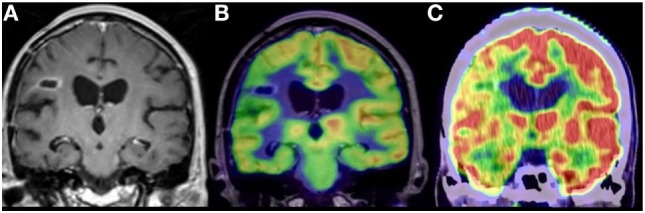
Anaplastic astrocytoma, status post surgery, radiation, and chemotherapy. **(A)** Contrast-enhanced coronal MR image demonstrating nodular enhancement about a right frontal operculum cavity. **(B)** Simultaneous co-registered positron emission tomography (PET)-MR image accurately demonstrating that the area of nodular enhancement has no associated elevated fluoro-2-deoxy-d-glucose (FDG) avidity. **(C)** Coronal image from a fused FDG PET-CT demonstrating no elevated FDG avidity in the region of nodular enhancement, although the anatomical resolution is limited compared to the PET-MR. No copyright permissions were required for use of these images.

Several limitations to PET imaging of gliomas should be addressed. Practically, the added cost, time, and radiation exposure of PET are hindrances for the routine addition of PET to MR imaging surveillance of gliomas. Furthermore, besides FDG, these PET tracers remain investigational and require access to radiochemistry laboratories and a cyclotron for ^11^C-compounds. Finally, PET imaging is limited by suboptimal spatial resolution; FDG uptake can be difficult to discern adjacent to areas of normal metabolically active cortex, and ^18^F-FDOPA can be difficult to discern adjacent to the basal ganglia. Indeed, one study comparing FDG-PET and MR perfusion found that FDG PET was less accurate in differentiating tumor from treatment-related changes (73). The advent of simultaneous PET-MRI scanners addresses some of these issues by reducing radiation exposure compared to a PET-CT, reducing imaging time by simultaneously acquiring the PET and MRI, and improving anatomical resolution for precise localization of metabolic uptake (Figures 3 and 4). Future studies will determine whether simultaneous PET-MRI significantly increases accuracy and changes management in the setting of brain tumor imaging. Until this added accuracy is demonstrated, FDG-PET will likely serve as a problem-solving tool when MR perfusion and spectroscopy are equivocal.

## Radiogenomics

Classically, the field of radiology has been primarily focused on correlating imaging and histopathological findings. However, oncologic diagnosis is quickly moving beyond traditional morphological approaches to molecular stratification; the traditional radiological–pathological correlation paradigm alone is no longer sufficient (74). In addition, while still the gold standard, numerous studies have shown that the histopathological classification of diffuse gliomas is prone to high inter-observer variation, correlates inconsistently with genetic markers, and imperfectly predicts clinical outcomes (75, 76).

**Figure 5:**
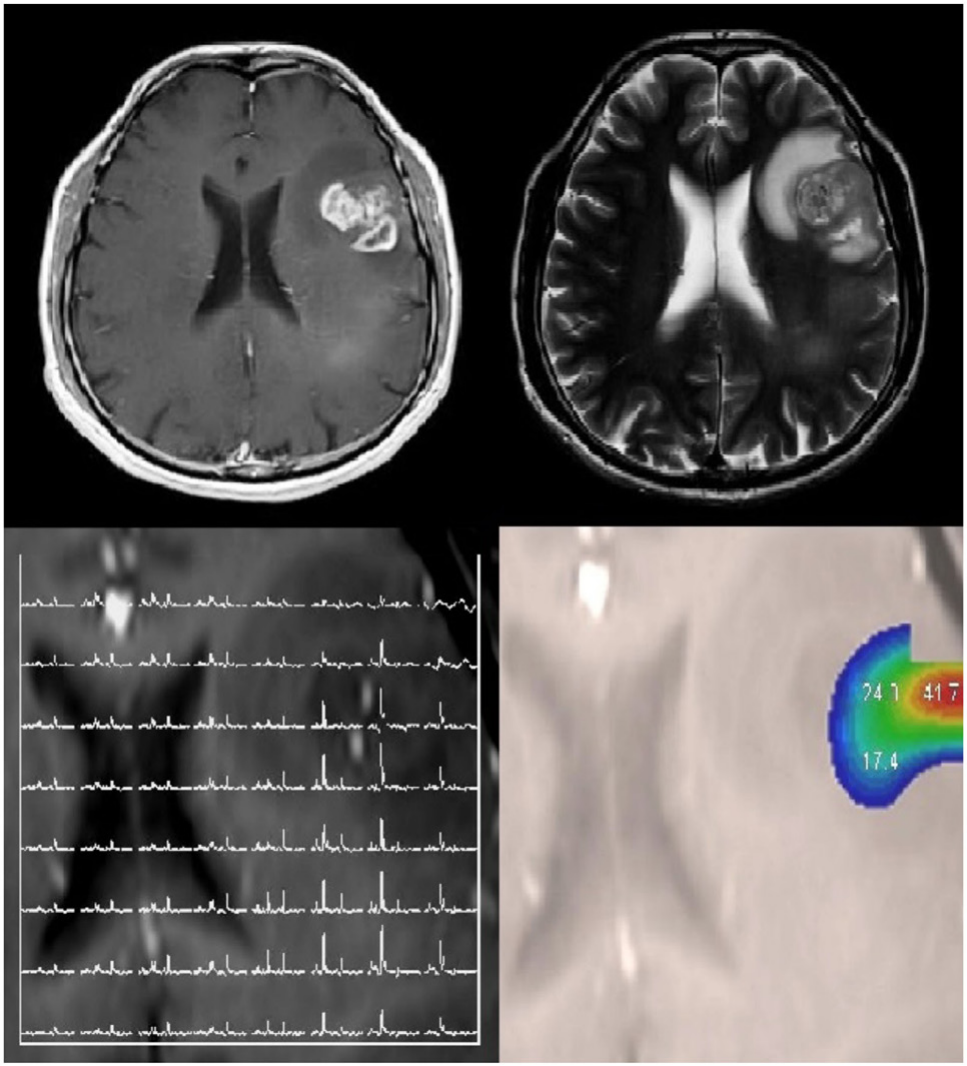
Radiogenomics involves correlating molecular alterations in tumors with conventional and advanced imaging findings. A patient with a left frontal glioblastoma, which was isocitrate dehydrogenase wild type, EGFR amplified, with phosphatase and tensin homolog loss, and a mutated TERT promoter. This lesion is shown on T1-weighted contrast-enhanced and T2-weighted images. Multivoxel MR spectroscopy and choline-to-*N*-acetylaspartate color maps are also shown. No copyright permissions were required for use of these images.

Radiogenomics is a promising new paradigm to bring clinical imaging into the molecular and genomics era, by identifying relationships between imaging features (imaging phenotypes) and molecular markers (Figure 5). The imaging and molecular data can then be integrated with clinical outcomes, such as overall survival, time to progression, or response to a particular drug therapy. Although imaging data cannot substitute for histopathologic findings, extracted quantitative imaging parameters from standard-of-care images, if correlated with genomic information, are able to highlight underlying physiologic, even subcellular processes (77). Imaging phenotypes of GBM obtained from routine clinical MRI studies can, therefore, be correlated with specific gene and microRNA expression signatures, serving as non-invasive surrogate markers of malignant genomic events and providing important information regarding diagnosis, prognosis, and optimal treatment (74, 78–81).

Understanding inter- and intra-tumoral heterogeneity in GBM is also important because both likely contribute to treatment failure (82, 83). Radiogenomics can potentially help tailor treatment strategy against intra-tumoral heterogeneity, which contributes to a tumor’s regional variations in metabolism, vasculature, oxygenation, and gene expression.

To date, several explorative studies in the field of radiogenomics have demonstrated the relationship between non-invasive imaging features such as contrast enhancement, invasiveness, location, volume, diffusion, and corresponding molecular characteristics.

In 2008, Diehn et al. first reported associations between GBM MR imaging, molecular phenotypes derived from DNA microarray analysis, and survival. Genes implicated in angiogenesis and tumor hypoxia, particularly vascular endothelial growth factor, were associated with the degree of contrast enhancement, and a high ratio of contrast enhancing tumor to necrosis correlated with epidermal growth factor receptor overexpression (84).

Promoter methylation of the DNA repair gene O^6^-methylguanine-DNA-methyltransferase (MGMT) is associated with improved survival following radiotherapy and chemotherapy in GBM patients. Levner and colleagues reported that texture analysis features extracted from conventional MRIs, including gray level patterns, pixel interrelationships, and spectral patterns, were predictive of MGMT methylation status. Using these features obtained from 59 patients, they were able to achieve an average accuracy of greater than 87% in predicting MGMT methylation status (85). A similar study from Drabycz et al. compared visual assessment to computer-derived texture analysis for prediction of MGMT methylation status and found that ring enhancement is significantly associated with an unmethylated MGMT promoter. The authors also reported that there were significant differences between methylated and unmethylated tumors in the T2-weighted images when analyzing tumor ROIs, however, not in entire tumor volumes. Spectral texture features extracted from T2-weighted images were more specific markers of methylation status than visual identification of ring-enhancement (65 versus 39%) but at a loss of sensitivity (79 versus 93% for spectral and visual textural features, respectively) (86). Additional discoveries have been made regarding genomic correlates of necrosis and tumor location. For example, Iliadis et al. reported that MGMT-methylated tumors had less preoperative tumor necrosis and improved progression-free survival (87). Ellingson et al. demonstrated an association between tumor location and the genetic profile of tumor precursor cells; for example, MGMT promoter methylated tumors were located more frequently in the left temporal lobe. The authors also observed that MGMT unmethylated GBMs were smaller in volume on T1-contrast enhanced and T2-FLAIR images than MGMT-methylated GBMs (88).

Transcriptomics is another area where imaging and genomic studies may overlap. Transcriptomics is the study of the complete set of RNAs (transcriptome) encoded by the genome of a specific cell at a specific time or under a specific set of conditions. Comparison of transcriptomes allows the identification of genes that are differentially expressed in distinct cell populations or in response to different treatments. Previous studies have identified four unique GBM transcriptomal subtypes named proneural, neural, classical and mesenchymal. Tumors with the proneural gene expression subtype, those with an isocitrate dehydrogenase-1 mutation, and tumors lacking the loss of the phosphatase and tensin homolog (PTEN) mutation have been reported to occur most frequently in the frontal lobe (88). In a study of mesenchymal GBM, Naeini et al. found that the ratio of volume of T2 hyperintensity to contrast enhancement and central necrosis was significantly lower in this subtype (89).

In another example of the value of transcriptomics, You et al. investigated leptomeningeal dissemination in GBMs, hoping to clarify underlying molecular profiles contributing to this disease phenotype. They performed integrative analysis of whole transcriptome sequencing (RNA-Seq) gene expression patterns in GBMs with leptomeningeal involvement on imaging. They found that SPOCK1, EHD2, SLC2A3, and ANXA11 were all more highly expressed with leptomeningeal dissemination compared to a control group without leptomeningeal spread. Among these genes, SPOCK1 activates PI3K/Akt signaling to block apoptosis and promote proliferation and metastasis (90).

In 2011, the NCI launched the Cancer Genome Atlas (TCGA) project to further efforts to incorporate molecular markers into the clinical care of GBM patients. One of the earliest large-scale studies using data from the TCGA was performed by Zinn et al., who correlated T2 FLAIR tumor volume with 13,628 genes and 55 microRNAs. The authors found that tumors with high T2 FLAIR volume were enriched with genes and microRNAs involved in cellular migration and invasion; specifically, the Periostin gene and miR-219 were believed to promote mesenchymal transition and invasion. In their cohort, patients with high levels of Periostin expression had decreased survival and more rapid disease progression (81). Another TCGA study found that lower-grade gliomas with an IDH mutation and 1p/19q codeletion had the most favorable clinical outcomes and were often localized to the frontal lobes. Nearly all lower-grade gliomas with IDH mutations and no 1p/19q codeletion had mutations in TP53 and ATRX. Furthermore, the lower grade gliomas without an IDH mutation had genomic aberrations and clinical behavior that were strikingly similar to those found in primary glioblastoma (11). Zinn et al. also used TCGA data from 35 patients, correlating ADC values with genomic profiles. Tumors were grouped based on high and low ADC in a ROI placed on a nonenhancing area of T2 FLAIR hyperintensity within 1 cm of tumor-enhancement borders. They found that the low ADC group had upregulation of BMI1 and Cyclin D1 and downregulations of YAP1 and E2F3 (91). BMI1 is a known regulator of stem-like states in cancer cells and is associated with migration, invasion, and poor prognosis (91, 92). Separately, Gutman et al. demonstrated that volumetric measures could predict GBM mutations such as TP53, NF1, EGFR, RB1, and platelet-derived growth factor receptor alpha (PDGFRA) (93).

Another group evaluated correlations between DSC MR Perfusion and SWI and key molecular characteristics in 152 patients with newly diagnosed GBM. Imaging features, including tumor volumes, volume ratios, apparent diffusion coefficients, cerebral blood flow, cerebral blood volume, and intratumoral susceptibility, were correlated with DNA methylation subgroups, MGMT promoter methylation status, and hallmark copy number variations (EGFR, PDGFRA, MDM4, and CDK4 amplification; PTEN, CDKN2A, NF1, and RB1 loss). After evaluating univariate associations they also implemented machine learning–based classification models. They found that there was no tumor location predilection for any of the assessed molecular parameters. Increased relative cerebral blood volume and cerebral blood flow within enhancing tumor were associated with EGFR amplification and CDKN2A loss. However, no single imaging parameter was able to predict key molecular features with high accuracy, limiting the clinical utility of these techniques at this time (94). Interestingly, Colen et al. reported sex-specific molecular profiles of cell death in GBM. They found that female patients showed significantly lower volumes of necrosis on MRI than male patients, but had significantly shorter survival. They suggested that cell death in female patients with GBM is associated with oncogenes such as MYC, while cell death in male patients is associated with TP53 activity (95).

The visually accessible Rembrandt images (VASARI) is a newer resource hoping to engender robust and reproducible MRI reads by validating data obtained from different sites. The VASARI scoring system includes 30 semantic descriptors of imaging features of brain tumors clustered by categories pertaining to lesion location, morphology of the lesion substance, morphology of the lesion margin, alterations in the vicinity of the lesion, and extent of tumor resection. This type of work is critical to standardize imaging descriptions of pathology, especially in the development of future natural image processing software. Using the VASARI scoring system, Colen et al. showed that GBM patients with specific invasive imaging signatures such as ependymal involvement, invasion of deep white matter tracts, and tumor extension across the midline had significantly decreased overall survival as well as increased MYC oncogene activation and inhibition of NFKBIA (96).

## Conclusion

Metabolic imaging such as spectroscopy and PET remain valuable tools whose information can guide clinical decision making. However, each suffer from drawbacks as discussed above and future work aimed at improving their sensitivity and specificity will enable better patient care. In particular, metabolic imaging that can herald impending malignant degeneration of lower grade tumors would be of incalculable value. Similarly, radiogenomics has remarkable potential to accelerate precision medicine, but it is still early in its evolution. Optimum protocols for image acquisition and reconstruction must be identified and standardized, and robust segmentation algorithms that require minimal operator input need to be developed. Furthermore, informatics databases must be generated that incorporate imaging features with medical and genetic data (82, 97). Machine learning algorithms will help evaluate the enormous amount of data originating from the addition of advanced imaging techniques such as diffusion, DCE/DSC MR Perfusion, and MRS (especially 2-HG). Also necessary will be the precise correlation of tumor biopsy sites with corresponding imaging voxels (91). Finally, statistical methods and study designs for ongoing radiogenomic studies may need to be standardized to allow maximal progress (98). Further clinical investigation with larger sample sizes from multiple centers will help test and validate new techniques in this exciting field (74).

## Author Contributions

CC and DS contributed to the MR spectroscopy portion of the manuscript. RR and RM contributed to the overall concept and writing of the manuscript.

## Conflict of Interest Statement

The authors declare that the research was conducted in the absence of any commercial or financial relationships that could be construed as a potential conflict of interest.
